# The Diagnostic Significance of Coapplying a Rabeprazole Test with the SF-36 for Gastroesophageal Reflux Disease

**DOI:** 10.1155/2013/419375

**Published:** 2013-02-28

**Authors:** Ying Chen, Feng Wang, Yuanxi Jiang, Chen Wang, Liwen Yao, Ping Wu, Yili Tong, Huihui Sun, Shuchang Xu

**Affiliations:** ^1^Department of Gastroenterology, Tongji Hospital, Tongji University School of Medicine, Shanghai 20065, China; ^2^Department of Gastroenterology, the Tenth People's Hospital of Shanghai, Tongji University School of Medicine, Shanghai 200072, China; ^3^Department of Private Ward, Tongji Hospital, Tongji University School of Medicine, Shanghai 200065, China; ^4^Department of Clinic Nutrition, Tongji Hospital, Tongji University School of Medicine, Shanghai 200065, China

## Abstract

Gastroesophageal reflux disease is a diversity disease that affects life quality of people in the world. Due to the complicated pathogenesis and variations in clinical manifestations, there is still no true gold standard for GERD diagnosis, and it is still difficult to diagnose this disease in some patients. The proton pump inhibitor's diagnostic test (the PPI test) is noninvasive, of low cost, tied to treatment, and widely accepted. Our aim is to evaluate the diagnostic significance of coapplying a rabeprazole test with the SF-36 for GERD in this study. Our study shows that the SF-36 in combination with the rabeprazole test can screen GERD patients and increase the sensitivity and specificity of GERD diagnosis through reference to the change in SF-36 score before and after the treatment (65 in the trial).

## 1. Introduction

Excessive gastroesophageal reflux can cause damage to the esophageal, throat, and even tracheal mucosa. Patients with gastroesophageal reflux disease (GERD) present with symptoms of heartburn, sour regurgitation, vomiting, onset retrosternal pain, and dysphagia. Bodily pain may influence mental status and is negatively associated with the patients' work, study, and social intercourse. GERD has become a critical digestive system disease that strongly influences the quality of life [1].

GERD diagnoses are primarily based on reflux symptoms, 24-hour esophageal pH monitoring, and endoscopy. However, these tests have limitations. NERD (nonerosive reflux disease) patients account for 60% to 70% of GERD patients [2] and have no signs of esophagitis in endoscopy. Therefore, negative endoscopic results cannot exclude GERD, which decreases the sensitivity of endoscopy [3]. Previously, 24-hour esophageal pH monitoring was used as the “gold standard” for GERD diagnosis. However, 25% of patients with typical reflux esophagitis (RE) have normal acid exposure [4, 5]. Therefore, the value of 24-hour pH monitoring for diagnosing GERD is decreased. Moreover, the invasiveness of this technique limits its application in clinical practice. In recent years, novel testing technologies and methods have been continuously developed, including the proton pump inhibitor (PPI) diagnostic test, the reflux questionnaire, and esophageal impedance monitoring, all of which have dramatically improved GERD diagnosis. However, due to the complicated pathogenesis and variations in clinical manifestations, there is still no true gold standard for GERD diagnosis, and it is still difficult to diagnose this disease in some patients.

The proton pump inhibitor's diagnostic test (the PPI test) is noninvasive, of low cost, tied to treatment, and widely accepted [6, 7]. It has been reported that the sensitivity of the PPI test is 70% to 80% and the specificity is 55% to 85% [8]. GERD's complicated pathogenesis, however, results in poor sensitivity in certain patients. Application of the PPI test for diagnosing these patients is limited, and other diagnostic methods are required.

The reflux questionnaire is convenient and economical within a certain range of sensitivity and specificity [9, 10]. Some studies illustrate that coapplying symptom scoring alongside the PPI test could increase specificity to 91% [11]. However, current questionnaire surveys have different focuses and vary in diagnostic values. Strong subjectivity, overly brief symptomatic scores, and few scoring levels make questionnaire surveys unable to sensitively reflect changes in health status and reach clinical requirements.

The 36-item short-form health survey (SF-36) is the most common method in the standardized measurement of the quality of life and has been widely used in a series of studies because of its excellent reliability, validity, sensitivity, and feasibility. Recently, the quality of life for GERD patients has been studied more and more [12–17].

In this study, we utilized a combination of the PPI test and the SF-36 to offer an important basis for GERD diagnosis, particularly for NERD patients. This approach increases the diagnostic efficiency and the cost-efficacy ratio of GERD diagnoses.

## 2. Materials and Methods

### 2.1. Subjects

A total of 90 patients were enrolled from September 2008 to December 2009. The inclusion criteria were as follows: (1) the patient visited due to epigastric discomfort, including sour regurgitation, vomiting, heart burn, and retrosternal pain, and was suspected to have GERD; (2) the patient was male or female, aged 18 to 70 years; (3) the patient was educated at least as far as elementary school and could read and fill out the questionnaire independently; (4) the patient had not been treated with nonsteroidal antiinflammatory drugs, PPI, H^2^-receptor antagonists, anticholinergic agents, antibiotics, or prokinetics in the four weeks prior to visiting; (5) the patient provided written informed consent; (6) an RDQ score ≥ 6 was taken as a basic criterion.

Exclusion criteria were as follows: (1) the patient could not tolerate endoscopy or 24-hour esophageal pH monitoring; (2) the patient had other digestive tract diseases and systemic diseases that could induce digestive discomfort, such as diabetes mellitus, systemic sclerosis, or nervous system diseases; (3) the patient had a history of gastroesophageal surgeries, esophageal stenosis, digestive ulcers, and esophageal or gastric tumors; (4) the patient was pregnant, nursing, or suffered from severe cardiac, hepatic, or renal insufficiency.

All the enrolled patients filled out the SF-36 questionnaire under the direction of trained gastroenterologists. They received endoscopy and 24-hour esophageal pH monitoring. Patients with esophageal erosion in the endoscopy were graded according to the Los Angeles (LA) classification. Patients with a positive result on one of the two measures (endoscopy or 24-hour esophageal pH monitoring) were considered to have GERD; otherwise, they were classified as non-GERD.

### 2.2. Methods

Ninety patients were randomly and double-blindly divided into Group A (*n* = 30, rabeprazole 20 mg b.i.d. for 2 weeks), Group B (*n* = 30, rabeprazole 10 mg b.i.d. for 2 weeks), and Group C (*n* = 30, placebo for the 1st week and rabeprazole 10 mg b.i.d. for the 2nd week). The drugs were taken orally twice a day, 15 to 30 minutes before meals. All drugs and placebos used in the study were provided by Xian-Janssen Pharmaceutical Ltd.

All the agents were delivered one week before the treatment. The SF-36 and RDQ measurement scales were administered before therapy, one week after therapy, and two weeks after therapy. 

## 3. Results

### 3.1. General Information

The ninety included patients (mean age 44.13 ± 12.71 years) consisted of fifty-nine men and thirty-one women (the male-to-female ratio approached 2 : 1), of which thirty-three (36.7%) showed negative results in endoscopy and fifty-seven (63.3%) had RE. They were classified according to LA: 31 for REA, 23 for REB, 3 for REC, and 0 for RED. No significant differences were noted between the three groups in sex, age, disease severity, lower esophageal sphincter pressure (LESP), upper esophageal sphincter pressure (UESP), and symptom index (SI) (*P* > 0.05) (Table 1).

### 3.2. Comparisons of SF-36 Scores before and One and Two Weeks after the Treatment

There were no significant differences among three groups in the pretreatment, one week and two weeks after treatment (*P* > 0.05). And there were significant differences between pretreatment and two weeks after treatment in each group (*P* < 0.05). Further analysis found that SF-36 scores showed significant differences between GERD and non-GERD patients in group A two weeks after treatment, but not between GERD and non-GERD patients before one and two weeks after the treatment in Groups B and C (*P* > 0.05) (Table 2).

### 3.3. Comparison of Improving SF-36 Scores between GERD and Non-GERD Patients

The differences were significant in improving SF-36 scores between GERD and non-GERD patients in Groups A and B (*P* < 0.05) but not significant between GERD and non-GERD patients in Group C (*P* = 0.085) after one week of therapy. Significant differences were noted between GERD and non-GERD patients in all three groups after two weeks of therapy (*P* < 0.05) (Table 3).

### 3.4. Comparison of Improvement Rate of SF-36 Scores between GERD and Non-GERD Patients after Treatment

There were significant differences in improvement rates for SF-36 scores between GERD and non-GERD patients in Group A (*P* = 0.006) but not significant differences between GERD and non-GERD patients in Groups B and C (*P* > 0.05) after one week of therapy; significant differences were noted between GERD and non-GERD patients in Group A (*P* = 0.037) but not in Groups B and C after two weeks of therapy (*P* > 0.05) (Table 4). 

### 3.5. Value of the SF-36 in Diagnosing GERD Prior to Treatment

**Figure 1:**
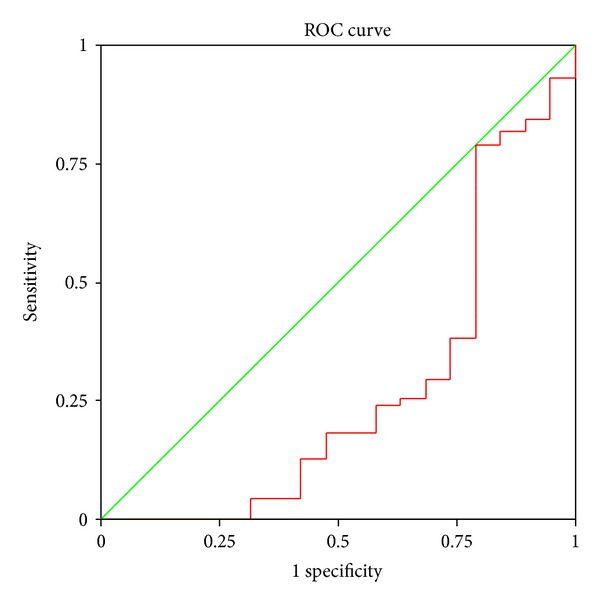
ROC of SF-36 scores prior to treatment.

A receiver operating characteristic (ROC) curve was plotted on the basis of pretreatment SF-36 scores. The area under the ROC (Az) was 0.27, indicating poor diagnostic value. Therefore, it was not suitable for diagnosing GERD (Figure 1).

### 3.6. Effects of Rabeprazole Dose and Treatment Course on Coincident Rate of the Rabeprazole Test

The scores decreased in group A (40 mg/day) and group B (20 mg/day). However, the differences in sensitivity, specificity, and coincident rate were not significant from the perspective of diagnostic efficacy (*P* = 0.095, 0.117, resp.). Significant differences were also not noted in overall coincident rate between one-week and two-week treatment in Group A (40 mg/day) and Group B (20 mg/day) (*P* = 0.688, 0.774, resp.) (Tables 5 and 6).

### 3.7. Value of Coapplying the Rabeprazole Test and SF-36 in Diagnosing GERD

**Figure 2:**
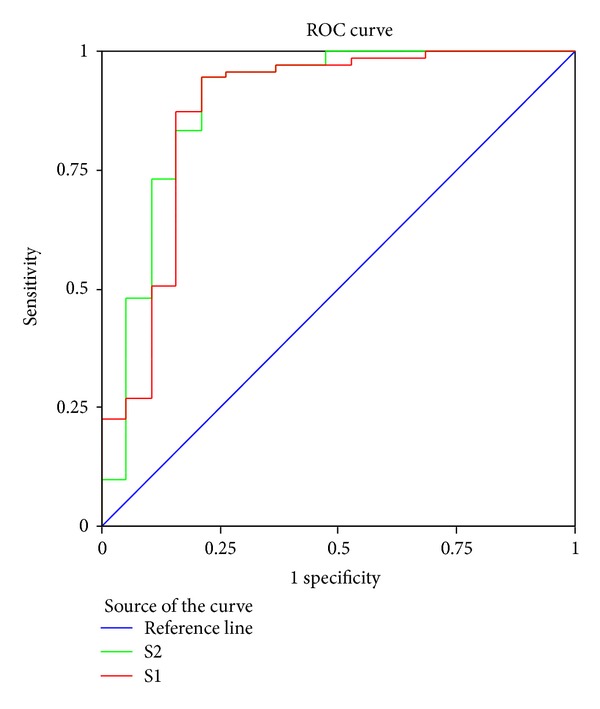
ROC of changes of SF-36 scores after one-week or two-week treatment.

**Figure 3:**
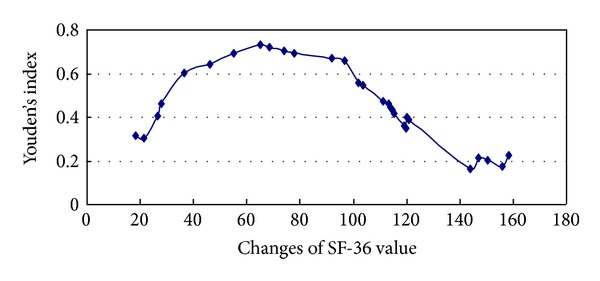
Changes of Youden's index in GERD patients.

The 95% confidence intervals (CI) of Az after one- and two-week treatment overlapped, illustrating that the diagnostic value after one week and two weeks did not differ significantly (Figure 2). Results were judged using different cut-off values according to the decreasing score within 1 week. Lastly, a score of 65 was taken as the cut-off value in line with the maximal principle of the Youden index (Figure 3), within which the sensitivity and specificity were optimal for the GERD screening.

### 3.8. Logistic Regression Analysis of Coapplying the Rabeprazole Test and SF-36

**Figure 4:**
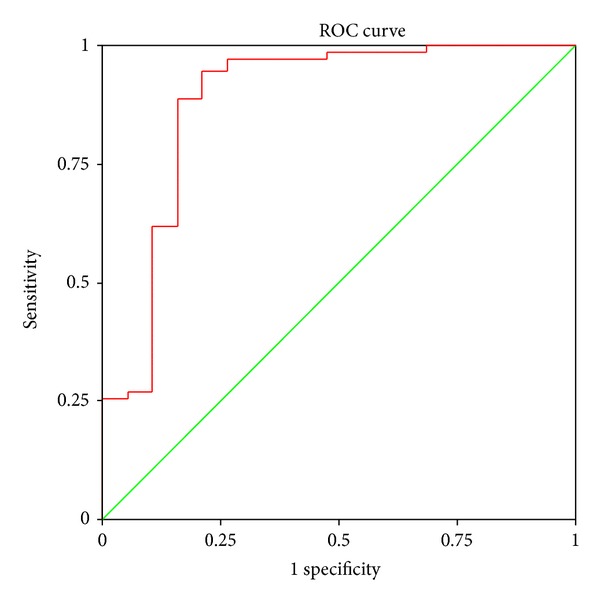
ROC curve for coapplying rabeprazole test and SF-36 in diagnosing GERD.

The area above the ROC was 0.884 (95% CI, 0.778–0.991, *P* < 0.001), illustrating that this score screening yields excellent reliability for integrative screening (Figure 4).

**Figure 5:**
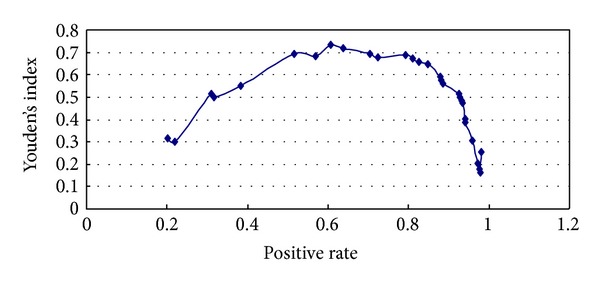
Youden's index for coapplying rabeprazole test and SF-36 in diagnosing GERD.

At *P* = 0.606, the Youden index was highest, and the sensitivity was negatively associated with the specificity. This value can be defined as the threshold for screening tests (Figure 5).

### 3.9. The Sensitivity and Specificity of the Rabeprazole Test Combined with SF-36 in Diagnosing GERD when *P* = 0.606

Based on the above results, rabeprazole 10 mg b.i.d. was applied in the PPI test. An increment of 65 score units from pretreatment to posttreatment was taken as the standard for a GERD diagnosis. Thus, of 71 patients with GERD, 67 were diagnosed with GERD and 4 were excluded from GERD; the false negative rate was 5.6%. Of 19 non-GERD patients, 4 were diagnosed with GERD and 15 were excluded from GERD; the false positive rate was 21.1% (Table 7).

## 4. Discussion

A randomized, double-blind, and controlled design was adopted in this trial. The physicians who were responsible for endoscopy, 24-hour esophageal pH monitoring, and administering the questionnaire survey were relatively independent. After tests, another physician performed the statistical analysis in order to guarantee the objectivity and validity of tests. 

Results showed that the 90 patients consisted of 59 males and 31 females, with the male-to-female ratio approaching 2 : 1. This ratio is similar to previous reports and may be due to histories of smoking, and drinking. Reportedly [17], drinking, smoking, obesity and overeating are major risk factors for GERD. No significant differences were noted in age, LESP, UESP, and SI among the three groups (*P* > 0.05), suggesting that patients in various groups were comparable after randomized and double-blinded grouping. 

Group A (40 mg/day for two weeks), Group B (20 mg/day for two weeks), and Group C (placebo for the 1st week and rabeprazole 20 mg/d for the 2nd week) were designed to investigate the effects of PPI in different doses and treatment duration on test results. Results indicate that SF-36 scores had no significant differences between pretreatment and after one or two weeks of treatment, which may be attributed to non-GERD patients mingling between all the groups. Therefore, GERD and non-GERD patients should be analyzed separately. When patients were grouped according to GERD and non-GERD diagnosis, differences in SF-36 scores were only noted between GERD and non-GERD groups after two weeks of treatment for Group A. The differences were not significant between GERD and non-GERD groups before and after one and two weeks of treatment in other groups. It is believed that SF-36 scores, the common disease scale, are affected not only by GERD itself but also the occupation, material status, family and social relationships, education, household income, and social class of patients. The Az was 0.27 in the SF-36 score ROC prior to the treatment, suggesting that SF-36 alone is a poor tool for diagnosing GERD in a primary care setting and is not sufficient to establish a GERD diagnosis. Further analysis showed that the differences were significant in improved SF-36 scores between GERD and non-GERD patients in Groups A and B (*P* < 0.05) but not significant between GERD and non-GERD patients in Group C (*P* = 0.085) after 1-week therapy; significant differences were noted between GERD and non-GERD patients among the three groups after two-week therapy (*P* < 0.05). However, statistical differences in improvement rate were not noted between GERD and non-GERD patients in Groups B and C after two-week treatment (20 mg/d), as the improvement rate is correlated with improved scores and basic scores, and the basic SF-36 scores are related not only to GERD itself but also to many other factors mentioned above. In Group A (40 mg/d), statistical differences in improvement rate are noted between GERD and non-GERD patients, which may be strongly related to the increase of improved scores. Improved scores and basic scores can both influence the improvement rate. Therefore, improvement rate is not regarded as a criterion of the PPI test in improving scores. An improved score, the difference in life quality before and after the treatment, represents the degree of improvement in life quality and can be affected only by a few factors. Therefore, the improved score can be taken as a criterion for the PPI test. Our results show that according to the Youden value principle, an improved score to 65 is most efficient for a GERD diagnosis. At this level, the diagnostic sensitivity was 94.4% and the specificity was 78.9%. If the improved score is boosted, the specificity increases but the sensitivity decreases; therefore, an SF-36 score increase of 65 is taken as the criterion for a positive PPI test result. 

In this trial, the diagnostic value of the rabeprazole test did not differ significantly according to duration; the diagnostic value can be considered approximately equivalent after one week of treatment and after two weeks of treatment. The diagnostic coincident rate is 73%, the sensitivity is 85.7%, and the specificity is 44.5% after a one-week administration of rabeprazole 10 mg twice per day. Significant differences in diagnostic sensitivity and specificity at the two-week mark are not noted compared to that at the one-week mark. Diagnostic efficacy is consistent and the expense increases significantly. Therefore, diagnostic administration for two weeks is unnecessary for judging results. There are no significant differences in the sensitivity and specificity of the 40 mg/day and 20 mg/day groups. Therefore, rabeprazole given as 10 mg b.i.d. for one week is optimum for the PPI test and has the added benefit of being less costly. Results of diagnostic tests with rabeprazole, as reported by Schenk et al. [18], show that the sensitivity, specificity, and negative predictive value were, respectively, 68%, 63%, and 68% in the trial group and 20%, 95%, and 83% in the control group. Johnsson et al. [19] conducted a trial with omeprazole (20 mg b.i.d.) and results showed that the one-week sensitivity was 75% and the specificity was 55%. Cho et al. [20] reported that if lansoprazole 30 mg was given as b.i.d. for two weeks, the diagnostic sensitivity and specificity were 77% and 56%, respectively, illustrating that as the diagnostic sensitivity and specificity increased, the total coincident rate was similar, but the expense rose significantly. Therefore, the two-week administration did little to improve diagnosis. If patients with reflux symptoms are given rabeprazole 10 mg b.i.d. for one week and their SF-36 score increases 65 units following treatment, they can be diagnosed as GERD. Further logistic regression analysis suggests that the diagnostic sensitivity was 94.4%, specificity was 78.9%, the coincident rate was 91.1%, the false negative rate was 5.6%, and the false positive rate was 21.1%.

The SF-36 consists of 3 major parts: functional status, health satisfaction, and total evaluation. It includes eight fields: physical function, physical responsibility, body pain, activation, social function, and emotional responsibility. The eight fields are classified further into physical component scales and mental component scales. The SF-36, a common scale, not only measures its own items but also investigates several specific problems as affecting factors when determining the quality of life with GERD. It comprises more contents than the relatively limited RDQ. Some GERD patients present primarily with extraesophageal symptoms such as coughing and throat discomfort, which strongly influence the quality of life. After treatment, patients improved and their life quality increased. Therefore, rabeprazole in combination with the SF-36 can make a diagnosis through a comparison of pretreatment and posttreatment scores. A common scale may be more helpful than a GERD-specific scale to clarify the reason for decline in the quality of life. 

The PPI test is a current diagnostic method for GERD. This study aims to determine the effectiveness of coapplying the PPI test and the SF-36 for GERD diagnosis. Rabeprazole is metabolized in nonenzymic fashion, with a longer half-life, more stable pharmacokinetics, and greater efficacy than the first generation of PPI. In clinical practice, administration of rabeprazole can improve reflux symptoms and the quality of life rapidly [21–23]. In this study, SF-36 scores increase significantly after administration of rabeprazole and differences are more significant than the pretreatment. These differences are induced by rabeprazole for an individual with the same specific problems and thus interference from specific problems can be excluded. Administration of the basic SF-36 seems to have no value for diagnosing GERD at the patient's initial visit. However, increase in the SF-36 score after rabeprazole treatment can be used for the cut-off value in the rabeprazole test and thus provides the preliminary quantitative criteria for the PPI test. Large-sample, multicenter trials are required to confirm this result in clinical practice.

In conclusion, our study shows that the SF-36 in combination with the rabeprazole test can screen GERD patients and increase the sensitivity and specificity of GERD diagnosis through reference to the change in SF-36 score before and after the treatment (65 in the trial). This not only reduces the expense of clinical diagnosis but also reduces the pain that might be inflicted for gastroscopy and pH monitoring. With this method, diagnosis and treatment can be performed concurrently to shorten diagnosis duration.

Certainly, more exact diagnostic criteria require more large-sample, multicenter, randomized control, and double-blinded studies.

## Figures and Tables

**Table 1:** Characteristics of three groups.

	Age (years)	LESP (mmHg)	UESP (mmHg)	SI (%)
A group	43.13 ± 13.11	15.90 ± 7.24	42.19 ± 13.17	49.09 ± 35.13
B group	48.03 ± 11.41	17.77 ± 7.40	48.63 ± 19.97	46.86 ± 33.90
C group	41.23 ± 13.62	17.85 ± 6.87	44.47 ± 20.49	54.47 ± 35.75

*P* value	0.109	0.496	0.385	0.687

**Table 2:** Comparisons of SF-36 scores before and one and two weeks after the treatment.

	Before treatment	One week after treatment	Two weeks after treatment	*P* value
A group	551.81 ± 102.90	645.87 ± 74.54	691.63 ± 66.23	<0.00
B group	480.49 ± 114.98	615.37 ± 84.75	650.98 ± 91.23	<0.00
C group	517.47 ± 100.14	623.82 ± 74.97	686.45 ± 53.46	<0.00

*P* value	0.058	0.302	0.064	

**Table 3:** Comparison of improving SF-36 scores between GERD and non-GERD patients after one-week and two-week therapy.

	One week after therapy			Two weeks after therapy		
			*P* value			*P* value
	GERD	Non-GERD		GERD	Non-GERD	
A group	129.76 ± 44.84	10.52 ± 20.19	*P* < 0.00	181.10 ± 79.09	43.26 ± 30.40	<0.00
B group	144.26 ± 60.37	73.88 ± 38.88	*P* = 0.031	144.26 ± 60.37	73.88 ± 38.88	0.038
C group	114.88 ± 49.77	72.22 ± 63.11	*P* = 0.085	185.59 ± 73.55	102.55 ± 98.78	0.028

**Table 4:** Comparison of improving SF-36 rate between GERD and non-GERD patients after one-week and two-week therapy.

	One week after therapy (%)			Two weeks after therapy (%)		
			*P* value			*P* value
	GERD	Non-GERD		GERD	Non-GERD	
A group	28.32 ± 25.78	2.26 ± 3.68	0.006	40.66 ± 43.87	8.24 ± 6.85	0.037
B group	36.22 ± 27.60	13.63 ± 6.38	>0.05	45.58 ± 35.25	16.76 ± 8.83	>0.05
C group	25.22 ± 17.43	16.13 ± 18.62	>0.05	42.35 ± 29.46	24.07 ± 31.62	>0.05

**Table 5:** Comparison of rabeprazole tests of different doses and treatment courses.

Administration duration	Dose	Sensitivity	Specificity	Coincident rate
One week	20 mg	85.7%	44.5%	73%
	40 mg	88.4%	63.4%	81%
Two weeks	20 mg	90.4%	33.3%	80%
	40 mg	92.3%	50.0%	83%

**Table 6:** Comparison of coincident rates of rabeprazole tests of different doses and treatment courses.

Dose	Treatment course		
	One week	Two weeks	*P* value
20 mg	73%	80%	0.688
40 mg	81%	83%	0.774

*P* value	0.095	0.117	—

**Table 7:** Sensitivity and specificity of the rabeprazole test combined with SF-36 in diagnosing GERD when *P* = 0.606.

Screening results	GERD		Non-GERD		Total	
	Number of patients	95% CI (%)	Number of patients	95% CI (%)	Number of patients	95% CI (%)
+	67	94.4 (89.1–99.7) Sensitivity	4	21.1 (2.7–39.4) False positive	71	94.3 (88.9–99.7) Positive predictive value
−	4	5.6 (0.2–10.9) False negative	15	78.9 (60.5–97.2) Specificity	19	67.8 (60.6–97.2) Negative predictive value

Total	71	100	19	100	90	91.1 (85.2–96.9) Coincident rate
